# Efficacy of comprehensive nursing intervention on nursing outcomes and prognostic quality of life in elderly patients with chronic heart failure

**DOI:** 10.12669/pjms.40.11.9241

**Published:** 2024-12

**Authors:** Xuefei Zhang, Shan Gao, Shuya Wei

**Affiliations:** 1 Xuefei Zhang, Department of Cardiovascular Medicine, Baoding No.1 Central Hospital, Baoding 071000, Hebei, China; 2 Shan Gao, Department of Cardiovascular Medicine, Baoding No.1 Central Hospital, Baoding 071000, Hebei, China; 3 Shuya Wei, Department of Cardiovascular Medicine, Baoding No.1 Central Hospital, Baoding 071000, Hebei, China

**Keywords:** Comprehensive nursing, Elderly, Chronic heart failure, Nursing outcome, Quality of life

## Abstract

**Objective:**

To explore the effect of comprehensive nursing intervention on nursing outcomes, self-efficacy, and quality of life in elderly patients with chronic heart failure (CHF).

**Method:**

A retrospective analysis was conducted on the clinical data from 120 elderly patients with CHF admitted to Baoding No.1 Central Hospital from June 2019 to June 2022. Patients were divided into a control group and an observation group based on different nursing methods (n= 60 each group). The clinical data of the two groups were analyzed, comparing changes in heart function, psychological status, and quality of life before and after intervention.

**Results:**

After intervention, both groups exhibited decreased scores on the Hamilton Anxiety Scale and the Hamilton Depression Rating Scale compared to the pre-intervention levels, with the observation group showing a significantly lower degree of decline compared to the control group during the same period (P<0.05, respectively). The overall compliance rate of medical adherence in the observation group was 85.00%, significantly higher than the 70.00% in the control group (P<0.05). After intervention, both groups exhibited improvement in left ventricular ejection fraction, left ventricular end-systolic diameter, and left ventricular end-diastolic diameter compared to the pre-intervention levels, with the observation group showing a more significant improvement than the control group (P < 0.05, respectively).

**Conclusion:**

For elderly patients with CHF, comprehensive nursing intervention may reduce negative emotions, improve sleep quality, self-care ability, and self-efficacy, and enhance patient compliance, quality of life, and heart function.

## INTRODUCTION

Chronic heart failure (CHF) is a clinical syndrome characterized primarily by a decline in ventricular filling and pumping function due to various causes. Patients typically manifest decreased cardiorespiratory function, fatigue, and fluid retention.^1^ CHF represents the terminal stage of various cardiac diseases and stands as a major contributor to cardiovascular disease-related mortality. The incidence of CHF is notably higher in the elderly population, with studies indicating a risk as high as 33% for males and 28% for females aged 55 and above.^2^ Hospitalized patients with CHF face a substantial one year mortality rate of up to 17%.^3^ Presently, CHF significantly impacts human health and quality of life, posing a formidable challenge within the realm of cardiovascular diseases.^4^,^5^ As the aging population in China continues to increase, the number of elderly patients with CHF is rapidly increasing. Furthermore, the incidence of CHF among the elderly is on the rise each year. Given that elderly individuals experience a gradual decline in organ function, often suffer from multiple underlying diseases, and have lower immune and resistance capabilities, CHF in the elderly becomes a complex and rapidly progressing condition. It has emerged as one of the primary causes of hospitalization and mortality among the elderly^6^ The treatment objectives for elderly CHF patients focus on alleviating clinical symptoms, improving cardiorespiratory function, and enhancing overall prognosis and quality of life.^7^ While medication remains the mainstay in CHF management, challenges such as prolonged treatment periods and recurrent disease exacerbations contribute to reduced patient treatment adherence.^8^ Recognizing the significant impact of effective nursing interventions on CHF outcomes, implementing comprehensive nursing measures in conjunction with pharmacological treatment has proven effective in improving cardiorespiratory function and enhancing the prognosis and quality of life of patients.^9^ In recent years, comprehensive nursing measures have gained widespread clinical application and demonstrated clear clinical advantages. In light of this, this study presented a retrospective analysis of the nursing intervention effects on elderly patients with CHF in Baoding No.1 Central Hospital who received comprehensive nursing interventions, to explore the effect of comprehensive nursing intervention on nursing outcomes, self-efficacy, and quality of life in elderly patients with chronic heart failure (CHF).

## METHODS

This was a retrospective study. In China, citizens over the age of 60 are defined as the elderly, one-hundred and twenty elderly patients with CHF admitted to Baoding No.1 Central Hospital from June 2019 to June 2022, were divided into an observation group (n=60) and a control group (n=60) according to different nursing intervention methods.

### Ethical Approval:

The study was approved by the Institutional Ethics Committee of Baoding No.1 Central Hospital (No.: 2023103; date: October 23, 2023), and written informed consent was obtained from all participants.

### Inclusion criteria:

- Individuals meeting the diagnostic criteria for CHF^10^;
- Absence of limb motor dysfunction;
- Age≥60 years;
- Patients and their family members were knowledgeable about the study, capable of normal communication, willingly participated in the research, and signed informed consent forms.

### Exclusion criteria:

- Individuals with organ failure or severe complications;
- Individuals with language, consciousness, or cognitive function impairments, making normal expression difficult;
- Individuals unable to cooperate or complete follow-up for any reasons.

### The control group received standard nursing care, which included:

- Psychological intervention: actively communicating with patients, clarifying their understanding of the disease, and guiding them to overcome negative thoughts, fostering a positive mindset in dealing with the illness;
- Health education: providing CHF-related disease knowledge to patients, offering guidance on medication usage, ensuring patients understand the importance of medication adherence for disease control, and enhancing compliance with prescribed medications;
- Guidance on regular check-ups: advising patients to undergo periodic examinations and guiding them in cardiorespiratory exercise.

### The observation group received comprehensive nursing intervention:

- Establishment of a comprehensive nursing intervention team: comprising two physicians, one head nurse, and two nurses, with all team members receiving training on knowledge related to elderly CHF and comprehensive nursing, and jointly creating a WeChat group and an official account for communication with patients, respectively;
- Psychological intervention: listening to patients’ inner thoughts, identifying the root causes of negative emotions, assisting patients in relieving negative emotions, building confidence in treating the disease, and enhancing patient compliance;
- Health education: conducting effective health education for patients, guiding patients to actively control risk factors, preventing acute CHF exacerbations, establishing medication supervision and follow-up platforms, sending daily reminders to patients via WeChat or public account, conducting regular follow-up supervision, and periodically reminding patients of follow-up appointments;
- Nutrition and exercise care: developing a dietary plan based on patients’ eating habits, adjusting patients’ dietary structure, providing reasonable dietary arrangements, formulating targeted exercise training plans based on the patients’ condition, and guiding and assisting patients in progressive rehabilitation training;
- Sleep and reinforced behavioral intervention: during hospitalization, guiding patients to maintain a positive mindset, administering sedative medications when necessary, maintaining a quiet and warm hospital environment, instructing patients to develop regular sleep habits, providing guidance on cultivating positive behaviors, encouraging patients to maintain good emotional states under appropriate daily routines, and advising patients on developing positive behavioral habits based on the ward environment and dietary structure;
- Post-discharge follow-up: regularly sharing CHF-related knowledge through the official account and WeChat group, periodically conducting WeChat group video sessions, with nurses explaining CHF-related precautions and addressing patient questions, and facilitating the sharing of diagnosis and treatment experiences by patients with high compliance and good recovery.

### Outcome measures:

### Psychological status:

The Hamilton Anxiety Scale (HAMA) and the Hamilton Depression Rating Scale (HAMD) were employed to assess the pre- and post-intervention negative emotions in both groups. The HAMA scale consists of 14 items, with a score of ≥ 7 indicating anxiety and a higher score representing more severe anxiety. The HAMD scale comprises 24 items, with a score of ≥ 8 indicating depression and a higher score suggesting more severe depression.

### Adherence to medical advice:

The two groups were compared for post-intervention adherence to medical advice, and adherence was categorized as follows: i) complete adherence: strict patient compliance, regular daily routine, proper diet, and regular check-ups with a basic understanding of disease-related knowledge; ii) partial adherence: taking medication on time and in the prescribed quantity under supervision, but having difficulty adhering to one or two aspects in the domains of health knowledge, healthy habits, and regular check-ups; iii) non-adherence: non-compliance with medication instructions, irregular diet and sleep patterns, poor knowledge of health, and irregular check-ups. Overall adherence rate = (complete adherence cases + partial adherence cases) / total cases × 100%.

### Sleep quality:

The Pittsburgh Sleep Quality Index (PSQI) was utilized to evaluate sleep quality post-intervention. The total score on the PSQI ranges from 0 to 21 points, with higher scores indicating poorer sleep quality.

### Self-care ability and self-efficacy:

The Exercise of Self-Care Agency (ESCA) questionnaire was utilized to assess patients’ self-care ability. The questionnaire comprises four dimensions with a total of 43 items, including self-care concepts, self-care responsibility, self-care skills, and health knowledge level. Each item is rated on a Likert 4-point scale, with a total score ranging from 0 to 172. A higher score indicates better self-care ability. Apart from this, the General Self-Efficacy Scale (GSES) was employed to evaluate self-efficacy. This scale consists of 10 items, each rated on a Likert 4-point scale, with a total score ranging from 10 to 40. A higher score indicates higher self-efficacy. (5) Quality of life and activities of daily living (ADL) assessment: The Stroke-Specific Quality of Life Scale (SS-QOL) was administered before and after the intervention to assess patients’ quality of life. The scale covers four domains: linguistic function, roles, cognition, and physical functions, with scores ranging from 0 to 20 and a higher score indicating a better quality of life. In addition, the ADL assessment tool was used to evaluate the ability to perform daily activities. The assessment covers activities such as toileting, eating, dressing, grooming, walking, and bathing, with a total score of 100 and a higher score indicating better self-care ability.

### Cardiac function:

Color Doppler ultrasound was employed before and after the intervention to compare cardiac function indicators between the two groups, including left ventricular ejection fraction (LVEF), left ventricular end-systolic diameter (LVESD), and left ventricular end-diastolic diameter (LVEDD). In this study, all the questionnaire was given to the participants for investigation, and then collected in about five minutes.

### Statistical analysis:

All data in this study were statistically analyzed using SPSS21.0 software. Quantitative data were presented as mean ± standard deviation (*χ̅*±*S*), and intergroup comparisons were examined using the t-test. Qualitative data were expressed as counts and percentages (%), and intergroup comparisons were assessed using the chi-square (χ²) test. P < 0.05 was considered statistically significant.

## RESULTS

There were no statistically significant differences between the two groups in general clinical data, including gender, age, body weight, body mass index (BMI), and disease course (all P > 0.05). Table-I. There were no significant differences in the HAMA and HAMD scores before intervention between the two groups (P > 0.05, respectively). After intervention, both groups showed a decrease in the HAMA and HAMD scores, with the observation group exhibiting a significantly lower degree of decrease compared to the control group (P < 0.05, respectively). Table-II.

**Table-I:** Comparison of general clinical data between the two groups

Group	n	Sex (n)		Age (years)	Body weight (kg)	BMI (kg/m²)	Disease course (years)

		Male	Female				
Observation group	60	35	25	69.48±4.92	83.12±7.12	28.50±3.93	10.15±2.80
Control group	60	37	23	69.02±5.07	83.49±6.56	29.50±3.83	10.40±3.42
*t/χ*²		0.139		0.511	0.303	1.404	0.439
*p*		0.709		0.610	0.763	0.163	0.662

**Table-II:** Comparison of the HAMA and HAMD scores before and after intervention in the two groups (*χ̅*±*S*).

Group	HAMA score		HAMD score	

	Before intervention	After intervention	Before intervention	After intervention
Observation group (n = 60)	15.83±0.83	5.08±0.46	16.12±0.42	6.17±0.62
Control group (n = 60)	15.90±0.73	5.67±0.57	16.20±0.61	6.68±0.50
*t*	0.468	6.143	0.879	5.032
*p*	0.640	0.000	0.381	0.000

The total adherence to medical treatment in the observation group was 85.00%, which was significantly higher than the 70.00% in the control group (P < 0.05). Table-III. Before intervention, there were no statistically significant differences in the PSQI, ESCA, and GSES scores between the two groups (all P > 0.05). After intervention, both groups showed a decrease in the PSQI score, with the observation group exhibiting a significantly lower degree of decrease compared to the control group (P < 0.05). Additionally, both groups showed an increase in the ESCA and GSES scores, and the improvement was more evident in the observation group compared to the control group (P < 0.05, respectively). Table-IV.Before intervention, there were no statistically significant differences in the SS-QOL and ADL scores between the two groups (both P > 0.05). After intervention, both groups showed a significant improvement in the SS-QOL and ADL scores, and the improvement was more evident in the observation group compared to the control group (P < 0.05, respectively). Table-V.

**Table-III:** Comparison of adherence to medical advice between the two groups [n(%)].

Group	Complete adherence	Partial adherence	Non-adherence	Total adherence rate
Observation group (n = 60)	31(51.67)	20(33.33)	9(15.00)	51(85.00)
Control group (n = 60)	24(40.00)	18(30.00)	18(30.00)	42(70.00)
*χ*²				4.021
*P*				0.045

**Table IV:** Comparison of sleep quality, self-care ability, and self-efficacy before and after intervention in the two groups (*χ̅*±*S*).

Group	PSQI score		ESCA score		GSES score	

	Before intervention	After intervention	Before intervention	After intervention	Before intervention	After intervention
Observation group	17.40±1.74	5.40±2.74	84.73±6.36	147.45±6.59	18.25±0.75	29.65±0.52
Control group	16.95±1.87	6.65±2.46	85.63±4.37	134.13±5.24	18.30±0.53	24.28±0.56
*t*	1.364	2.629	0.903	12.243	0.421	54.895
*p*	0.175	0.010	0.368	0.000	0.674	0.000

**Table-V:** Comparison of the SS-QOL and ADL scores before and after intervention in the two groups (*χ̅*±*S*).

Group	SS-QOL score								ADL score	

	Physical function		Linguistic function		Roles		Cognition			

	Before intervention	After intervention	Before intervention	After intervention	Before intervention	After intervention	Before intervention	After intervention	Before intervention	After intervention
Observation group	4.72±0.45	10.85±0.88	5.90±0.77	14.97±1.18	5.75±0.47	15.48±0.93	7.77±0.46	18.47±1.02	58.75±6.99	84.33±6.73
Control group	4.53±0.68	7.48±0.91	5.63± 0.92	11.63±1.22	5.60±0.69	11.45±1.05	7.65±0.71	14.40±1.20	58.42±6.92	80.25±9.50
*t*	1.744	20.591	1.718	15.217	1.383	22.297	1.066	20.070	0.263	2.717
*p*	0.084	0.000	0.089	0.000	0.169	0.000	0.288	0.000	0.793	0.008

Similarly before intervention, there were no statistically significant differences in the levels of LVEF, LVESD, and LVEDD between the two groups (all P > 0.05). However, after intervention, both groups showed improvement in LVEF, LVESD, and LVEDD compared to the pre-intervention levels, and the improvement was more evident in the observation group compared to the control group (P < 0.05, respectively). Table-VI.

**Table-VI:** Comparison of cardiac function indicators before and after intervention in the two groups (*χ̅*±*S*).

Group	LVEF		LVESD		LVEDD	

	Before intervention	After intervention	Before intervention	After intervention	Before intervention	After intervention
Observation group	41.57±4.17	58.30±3.90	55.72±3.82	42.57±3.66	59.72±3.81	46.62±3.72
Control group	41.73±3.90	53.28±3.44	55.12±3.46	48.52±3.58	59.67±3.71	54.25±3.56
*t*	0.226	7.470	0.902	9.004	0.073	11.479
*P*	0.822	0.000	0.369	0.000	0.942	0.000

## DISCUSSION

The results of this study showed that, following intervention, the observation group’s HAMA, HAMD, PSQI, ESCA, and GSES scores were all superior to those of the control group (P < 0.05, respectively). In other words, implementing comprehensive nursing care for elderly patients with CHF helps them understand and comprehend CHF-related conditions. Additionally, it alleviates negative psychological states, leading to improved mental well-being, relaxation of body and mind, and subsequently enhancing the quality of sleep. Effective elimination of negative psychological states and insomnia allows patients to face the disease and life with a more positive attitude. Consequently, patients improve their self-care ability and self-efficacy.^11^,^12^ In this study, the degree of improvement in compliance with medical advice after intervention in the observation group was also superior to that of the control group (P< 0.05). This indicates that patient cooperation is higher after implementing comprehensive nursing care. This might be explained by the fact that under the comprehensive nursing care model, patients’ awareness of CHF is enhanced, negative psychological states are effectively eliminated, and sleep quality improves.^13^,^14^

The treatment principles for CHF aim to restore cardiac function and improve patients’ quality of life.^15^ Quality of life reflects the experiences brought about by a patient’s survival status related to expectations, purposes, reference standards, and matters of concern. Currently, quality of life has become an important indicator for assessing the condition and treatment efficacy in elderly patients with CHF.^16^,^17^ The study showed that implementing a comprehensive nursing care model for elderly patients with CHF can increase blood flow, reduce peripheral vascular resistance, inhibit sympathetic nervous system activity, promote cardiac function recovery, and improve quality of life.^18^,^19^

Comprehensive nursing models, starting from the patient’s individual circumstances, offer systematic, standardized, and comprehensive nursing care. These models can eliminate adverse factors hindering disease recovery, precisely guide nurses in implementing care measures, alter patients’ health perceptions, address rational factors, alleviate negative psychological states, enhance patient compliance, and provide effective care in a comprehensive manner. This approach improves patients’ self-care ability, inhibits disease progression, promotes the restoration of exercise capacity, improves peripheral circulation, and enhances cardiac pumping function.^20^

### Limitations:

It includes a modest sample size, single-center design, and short follow-up time. In the future, it is necessary to expand the sample size and extend the follow-up period to further validate the results of this study.

## CONCLUSIONS

Implementing comprehensive nursing care intervention for elderly patients with CHF may be an effective nursing approach to enhance patient compliance and self-care ability, alleviate negative psychological states, and improve sleep quality, thereby improving cardiac function and quality of life.

### Authors’ Contributions:

**XZ:** Carried out the studies, data collection, and drafted the manuscript,

**SG SW:** Performed the statistical analysis and participated in its design, Did review.

All authors read and approved the final manuscript and are responsible and accountable for the accuracy or integrity of the work.
